# Contribution of the Paraoxonase-2 Enzyme to Cancer Cell Metabolism and Phenotypes

**DOI:** 10.3390/biom14020208

**Published:** 2024-02-09

**Authors:** Roberto Campagna, Emma Nicol Serritelli, Eleonora Salvolini, Valentina Schiavoni, Monia Cecati, Davide Sartini, Valentina Pozzi, Monica Emanuelli

**Affiliations:** 1 Department of Clinical Sciences, Polytechnic University of Marche, 60126 Ancona, Italy; r.campagna@univpm.it (R.C.); emmaserritelli@gmail.com (E.N.S.); e.salvolini@univpm.it (E.S.); v.schiavoni@pm.univpm.it (V.S.); moniacecati@gmail.com (M.C.); v.pozzi@univpm.it (V.P.); m.emanuelli@univpm.it (M.E.); 2 New York-Marche Structural Biology Center (NY-MaSBiC), Polytechnic University of Marche, 60131 Ancona, Italy

**Keywords:** paraoxonase-2 enzyme, cancer cell phenotype, tumor biomarker

## Abstract

Paraoxonase-2 (PON2) is a ubiquitously expressed intracellular protein that is localized in the perinuclear region, the endoplasmic reticulum (ER), and mitochondria, and is also associated with the plasma membrane. PON2 functions as an antioxidant enzyme by reducing the levels of reactive oxygen species (ROS) in the mitochondria and ER through different mechanisms, thus having an anti-apoptotic effect and preventing the formation of atherosclerotic lesions. While the antiatherogenic role played by this enzyme has been extensively explored within endothelial cells in association with vascular disorders, in the last decade, great efforts have been made to clarify its potential involvement in both blood and solid tumors, where PON2 was reported to be overexpressed. This review aims to deeply and carefully examine the contribution of this enzyme to different aspects of tumor cells by promoting the initiation, progression, and spread of neoplasms.

## 1. Introduction

### 1.1. Paraoxonase-2 Enzyme and Oxidative Stress

**Figure 1:**
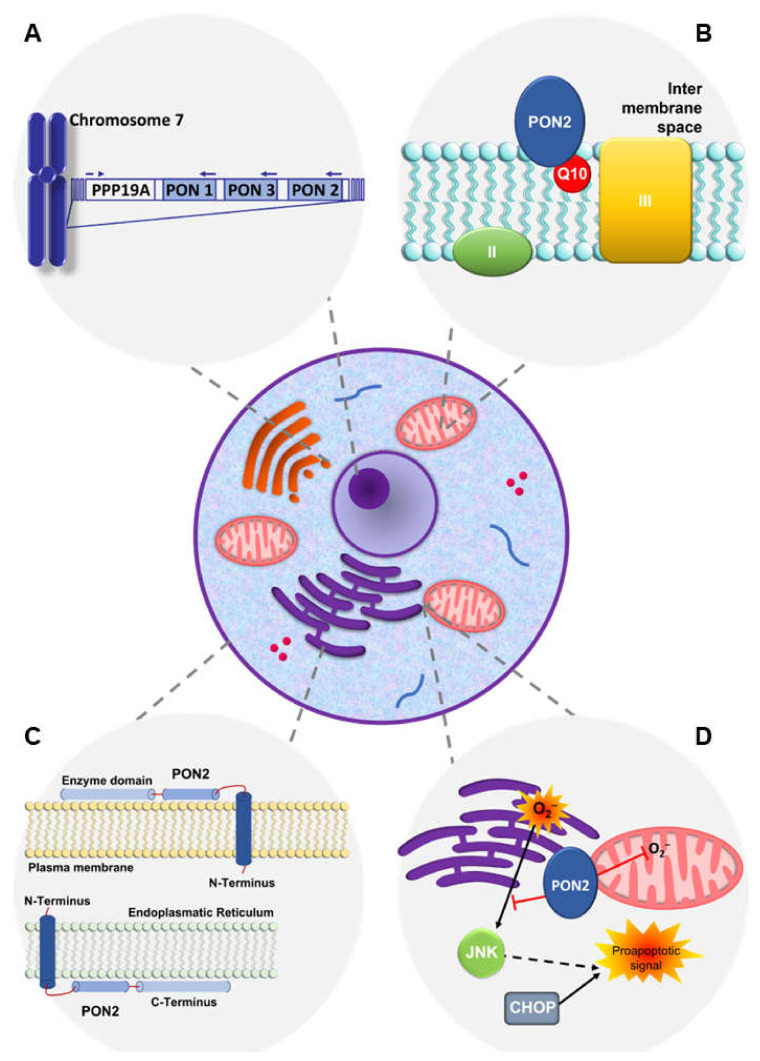
Intracellular overview of human PON2. (**A**) Chromosomal localization of the gene, and (**B**) association of the enzyme with mitochondria, (**C**) plasma membrane, and (**C**) endoplasmic reticulum. (**D**) Anti-apoptotic role of PON2 is mediated by its ability to counteract ROS-related oxidative stress.

The paraoxonase-2 (*PON2*) gene belongs to a multigene family that includes two other members, paraoxonase-1 (*PON1*) and paraoxonase-3 (PON3), with their names assigned according to the order of their discovery. The genes for these three paraoxonases (*PONs*) contain nine exons of approximately the same length, which are located close to one another, forming a defined cluster on the long arm of human chromosome 7, between regions q22.3 and q23.1 (Figure 1A) [1,2]. The *PON* members exhibit significant structural homology in mammals in terms of their nucleotide and amino acid sequences. In particular, *PON1*, *PON2*, and *PON3* share around 70% and 60% identity at the nucleotide and amino acid levels, respectively, within a given mammalian species, while each gene/protein displays 80 to 90% sequence identity between mammalian species [3]. Based on the structural homology, the evolutionary distance among *PON* family members has been explored, which revealed that *PON2* appears to be the oldest representative of the gene cluster, followed by *PON3* and *PON1* [4,5].

Transcription of the *PON2* gene generates two mRNA splice variants, each leading to the expression of a different protein, named PON2-iso1 (354 amino acids in length) and PON1-iso2 (342 amino acids in length), yielding bands of approximately 40 kDa. Both protein isoforms undergo post-translational modifications in terms of glycosylation, where the addition of mannose-type sugar residues at different positions is responsible for the expression of proteins reaching a molecular mass of 43 kDa [6,7]. Further functional analyses carried out to determine the impact of these glycosylation patterns on enzyme activity revealed that a cysteine-to-serine replacement at codon 311 in recombinant PON2 resulted in normal protein production and localization but altered glycosylation and significantly reduced lactonase activity toward *N*-3-oxododecanoyl homoserine lactone (3OC12-HSL) in Chinese hamster ovary (CHO) cells [8]. Moreover, the lack of glycosylation in both asparagine residues at positions 254 and 323 abolished 3OC12-HSL lactonase activity in EA.hy 296 endothelial cells [6].

Concerning enzyme activity, PON1 is able to hydrolyze various toxic oxon metabolites (paraoxonase activity) of a number of organophosphorus insecticides (e.g., parathion) and some nerve gases, such as sarin and soman. Through its arylesterase activity, PON1 also hydrolyzes aromatic esters, with aromatic esters of acetic acid being the most commonly used substrates. More recently, PON1 was also found to have lactonase activity, which enables the enzyme to catalyze the hydrolysis reaction of a variety of aromatic and aliphatic lactones (e.g., dihydrocoumarin), as well as the reverse reaction (lactonization) against hydroxycarboxylic acids. While PON2 and PON3 display very low or absent paraoxonase and arylesterase activity, they share the same ability to hydrolyze lactones as PON1 [2,6,8,9,10,11].

In humans, PON1 and PON3 are mainly expressed in the liver, and upon expression, they undergo secretion and associate with high-density lipoprotein (HDL) in the circulation and prevent low-density lipoprotein (LDL) oxidation due to reactive oxygen species (ROS) [1]. On the contrary, PON2 displays a wider expression pattern and is detectable in several organs, such as the heart, kidney, liver, lung, placenta, small intestine, spleen, stomach, and testis, as well as in endothelial cells [12,13,14]. The enzyme localizes within cells, notably in the perinuclear region, where it associates with the endoplasmic reticulum (ER) and nuclear envelope, as well as the mitochondria and plasma membrane (Figure 1B,C); thus, together with other intracellular antioxidant enzymes and systems, it protects cells from oxidative stress damage [7,12,15,16].

Within vascular endothelial cells, a considerable amount of PON2 was found to be associated with the ER (Figure 1C). In this context, PON2 enzyme activity was able to reduce the overwhelming levels of ROS production and release associated with the onset and progression of atherosclerosis, a vascular disorder characterized by biochemical events leading to severe ER stress and the activation of the unfolded protein response (UPR) pathway, which, in turn, may induce activation of the apoptotic executioner caspase-3. Therefore, PON2 upregulation, induced as a compensatory response to atherogenesis, may result in lower ER-associated oxidative stress, with consequent inactivation of UPR-induced apoptosis [7].

A further antiatherogenic effect was found to be exerted by the enzyme when it is localized at the plasma membrane (Figure 1C). In this location, PON2 behaves as an integral membrane protein, known as a single-pass type II transmembrane protein, with a single transmembrane hydrophobic N-terminal domain and a C terminus facing the extracellular compartment, which is responsible for retaining the enzyme activity and suppressing lipid peroxidation following extracellular oxidative stress. Signals associated with intracellular calcium release following surrounding oxidative stress induce PON2 translocation from the ER to the plasma membrane, where the N-terminal domain is used as an anchor, while the C terminus localizes extracellularly to counteract lipid peroxidation [14].

PON2 further contributes to lowering ROS production and oxidative stress associated with the development of atherosclerosis when it is located in the mitochondria (Figure 1B). Within these organelles, the enzyme is bound to the inner membrane, where it interacts with coenzyme Q10, leading to reduced release of superoxide anion from complex I and complex III as a consequence of oxygen leakage from the electron transport chain (ETC) [16,17].

### 1.2. Oxidative Stress, Inflammation, and Cancer

Oxidative stress is generically defined as an imbalance between ROS production and antioxidant defense systems, establishing a condition that favors the first over the second. The vast majority of ROS are produced within mitochondria during the series of redox processes associated with the ETC, the latter featuring the last stage of cellular respiration aimed at ATP synthesis. At the end of the ETC, oxygen acts as the final electron acceptor by combining with protons to produce water. However, about 1–2% of molecular oxygen escapes this physiological pathway and is converted into ROS. Molecular oxygen may undergo one-electron reduction, thus forming an intermediate superoxide anion, which in turn can be converted into hydrogen peroxide by superoxide dismutase (SOD) catalysis. Further Haber–Weiss or Fenton reactions ultimately generate highly reactive hydroxyl radicals [18,19].

Conditions associated with enhanced ROS formation can lead to impaired mitochondrial function and negatively affect cell viability. Based on the impact and extent of ROS-induced injury, specific pathways can be activated for damage repair or cell death triggering. While hydrogen peroxide can easily diffuse into the cytosol, superoxide anion is prevented from crossing biological membranes and, upon its generation, is released into the mitochondrial intermembrane space [20,21,22]. At this site, it can be converted into hydrogen peroxide by SOD, scavenged by cytochrome C with consequent activation of the apoptotic pathway, or diffused into the cytosol through pores of the outer mitochondrial membrane [23].

Although mitochondria appear to be the most powerful intracellular source of ROS, they are also provided with fundamental antioxidant defense systems, including enzymes and small peptides, such as glutathione (GSH). In addition to SOD, which can convert superoxide anion into hydrogen peroxide, GSH-linked enzymes like glutathione peroxidase 1 (Gpx1) and 4 (Gpx4) are involved in the reduction of hydrogen peroxide to water and lipid hydroperoxides, respectively [18].

Together with impaired mitochondrial respiration, the effects of ROS on cellular functions also include DNA damage, oxidation of proteins and lipids (peroxidation), and stimulation or inhibition of cell proliferation, until the induction of cell death, thus placing mitochondria at the center of apoptosis regulation [18].

ROS seem to be involved in the etiopathology of a wide range of neoplastic and non-neoplastic diseases that are characterized by the persistence of chronic inflammation. In particular, extensive and continued ROS-related oxidative stress can lead to the onset of chronic inflammation, which in turn can promote the development of many chronic cardiovascular, neurological, and pulmonary diseases, as well as diabetes, which is also associated with increased cancer risk [24]. Under physiological conditions, antioxidants outbalance pro-oxidants, while under oxidative stress conditions, pro-oxidants overwhelmingly prevail over antioxidants, which can lead to many inflammatory diseases, including cancer [25,26,27]. Inflammation-induced carcinogenesis is associated with a variety of molecular and phenotypic traits that contribute to cancer initiation, progression, and spread, such as genomic instability, epigenetic events leading to altered gene expression patterns, enhanced cell proliferation, resistance to apoptosis, neovascularization, metastatic invasion potential, and chemo- and radioresistance [24].

Previously reported data extensively illustrate the pivotal role played by the PON2 enzyme in reducing intracellular ROS production and counteracting the damage induced by oxidative stress at different sites, such as the mitochondria, ER, and plasma membrane. Since oxidative stress associated with mitochondrial dysfunction is implicated in the development of many inflammatory-based diseases, including atherosclerosis and cancer, it is conceivable that the anti-inflammatory effects exerted by PON2 in mitochondria and other organelles can somehow positively influence both the etiopathogenesis and progression of these disorders [28]. The enzyme’s ability to promote carcinogenesis, independently from its lactonase activity, stems from its capacity to dampen the detrimental effects of an overwhelming imbalance between pro-oxidant and antioxidant forces, thus allowing tumor cells to survive, proliferate, and spread.

The aims of this review were to provide an overview of the studies available in the scientific literature reporting altered PON2 expression in neoplasms, and to explore/reveal the involvement of enzymes in molecular mechanisms and cellular events featuring tumor cells.

## 2. PON2 and Cancer

The first studies exploring PON2 expression in solid cancers (tumor versus normal tissue) were carried out through cDNA microarray analysis and revealed enzyme upregulation in hepatocellular carcinoma and pancreatic adenocarcinoma, as well as in genitourinary neoplasms such as kidney, bladder, prostate, testicular, and endometrial cancers [29,30,31]. Further analyses performed at the protein level partly confirmed these data and allowed the detection of PON2 overexpression in lung and thymus cancers [31,32]. Moreover, significantly elevated enzyme levels were found in acute lymphoblastic leukemia (ALL), chronic myeloid leukemia (CML), and non-Hodgkin lymphoma [31,33,34,35].

While antioxidative and antiatherogenic [36,37] effects triggered by PON2 have been extensively elucidated and consolidated, data demonstrating its involvement in molecular processes that negatively influence programmed cancer cell death by inhibiting the intrinsic apoptotic pathway were recently reported following in vitro studies carried out in immortalized human vascular endothelial EA.hy 926 cells [32]. Intrinsic apoptosis is known to be driven by Bcl-2-activated proteins that are able to open the mitochondrial pores, thus determining the release of cytochrome C into the cytoplasm. Upstream intramitochondrial oxidative stress regulates this cascade of events by inducing cardiolipin peroxidation, with consequent disruption of its binding with cytochrome C. The anti-apoptotic function of PON2 is based on its ability to bind coenzyme Q10 and decrease superoxide anion formation, thus reducing cardiolipin peroxidation and cytochrome C release. PON2-mediated inhibition of intrinsic apoptosis is also associated with the enzyme’s capacity to counteract ER oxidative stress leading to activation of the UPR pathway. Indeed, PON2 exerts its protective effect against UPR-related apoptosis through the negative modulation of JNK signaling, which, in turn, reduces the induction of the pro-apoptotic CHOP transcription factor (Figure 1D) [31].

In light of those early discoveries, it was reasonable to hypothesize that enzyme overexpression in cancer might represent an adaptive strategy of tumor cells to escape the effects of oxidative stress induced by chemotherapeutic drugs. The induction of PON2 upregulation in EA.hy 926 cells treated with the anthracycline doxorubicin was associated with decreased intracellular ATP levels, as well as caspase-3 activation. In addition, apoptosis induced by treatment with the chemotherapeutic agent staurosporine or actinomycin D was significantly reduced in EA.hy 926 cells upon PON2 knockdown (Figure 1D) [32].

In addition to apoptosis and bioenergetics, which were first identified as molecular and biochemical aspects potentially regulated by PON2 overexpression, further cellular pathways were discovered to be affected by enzyme dysregulation featuring cancer cells. It was demonstrated that PON2 upregulation can positively influence different processes associated with intracellular metabolism, such as glucose intake and consumption through glycolysis (and not by the pentose phosphate pathway), as well as the tricarboxylic acid cycle (TCA) and pyrimidine nucleotide biosynthesis. A high intracellular glucose level together with downstream intermediates of the glycolytic and TCA pathways contribute to the inhibition of AMP-activated protein kinase (AMPK), which in turn promotes tumor growth and metastasis [38,39].

In the last decade, many studies have extended the analysis of PON2 involvement to other neoplasms, trying to deeply investigate its potential contribution to cancer cell aggressiveness.

### 2.1. PON2 and Oral Cancer

Western blot and immunohistochemistry analyses carried out in two separate groups of tumor and adjacent normal tissue samples obtained from cohorts of patients with oral squamous cell carcinoma (OSCC) revealed markedly increased PON2 levels in cancer compared with healthy-looking oral mucosa. In addition, a significant inverse correlation was found between PON2 expression and tumor size, highlighting potential enzyme involvement in the early phases of oral tumorigenesis. Further investigations of transcriptome data available in the Cancer Genome Atlas (TCGA), a public online database, demonstrated that PON2 expression was inversely related to the overall survival of OSCC patients, suggesting the promising prognostic value of the enzyme in association with this neoplasm [40]. These results agree with those of a previously published study that evaluated enzyme levels in the tumor and normal oral mucosa of OSCC patients who were followed for 3 years after surgical treatment. The reported data demonstrated significantly different PON2 expression (tumor versus normal tissue) in patients who experienced disease relapse compared with those who had relapse-free survival, thus suggesting that the enzyme may have interesting predictive power for disease progression [41].

Subsequent studies were performed on the HSC-3 and HOC621 OSCC cell lines, in which PON2 gene silencing was achieved and the impact on tumor cell phenotype was explored. The results revealed that enzyme downregulation significantly decreased cell proliferation and viability, enhanced sensitivity to treatment with the chemotherapy drug cisplatin (CDDP), and led to apoptosis activation, triggered by caspase-3, -8, and -9. Data obtained through Fourier transform infrared microspectroscopy clearly showed that upon CDDP treatment, there was significantly greater oxidative damage associated with lipids and proteins in PON2-silenced cells with respect to controls. Considering the anti-apoptotic effect exerted by PON2, it is reasonable to expect that modulating enzyme expression could greatly influence the response of cancer cells to conventional CDDP-based treatment based on the drug’s mechanism of action. Intracellular CDDP behaves as a potent electrophilic agent, rapidly reacting with nitrogen atoms of nucleic acid bases and inducing structural DNA damage with consequent cell cycle arrest and apoptosis activation. In addition, the apoptosis-mediated cytotoxic effect generated by CDDP is induced by oxidative stress. In light of these observations, it is conceivable that PON2 knockdown is associated with decreased cell viability as well as enhanced induction of apoptosis of OSCC cells treated with CDDP [42].

In addition to chemoresistance, the role of the enzyme was also investigated in molecular mechanisms that promote cell resistance to radiotherapy. In a study by Krüger et al. [43], PON2 was reported to be variably expressed in different OSCC tissue samples and cell lines, with the lowest enzyme levels in PCI-13 cells and the highest in SCC-4 cells. Radiation treatment induced enzyme expression as well as caspase-3/-7 activation, generating a response that was inversely related to the basal/endogenous protein level of each cell line. Accordingly, PON2 knockdown was associated with significantly increased activation of radiation-induced apoptosis of OSCC cell lines, strongly suggesting that the enzyme has the ability to protect cells from apoptotic damage caused by radiation treatment [43]. Analyses focused on revealing the molecular mechanisms underlying the transcriptional activity of the PON2 gene in OSCC cells demonstrated that the induction of enzyme expression is positively regulated by lymphoid enhancer-binding factor 1 (Lef-1), which serves as an activator of the Wnt/GSK3β/β-catenin pathway. Consistent with these findings, endogenous Lef-1 activity was found to be markedly higher in SCC-4 than PCI-13 cells, which was responsible for the different PON2 levels detected in the two cell lines [41].

### 2.2. PON2 and Skin Cancers

Among non-melanoma skin cancers (NMSCs), immunohistochemical expression of PON2 was first analyzed in the most recurrent basal cell carcinoma (BCC) by using surrounding normal-looking tissue margins as controls. In the same study, immunohistochemistry was also carried out to evaluate enzyme levels in melanoma samples and dermal melanocytic nevi. In addition, the existence of a relationship between enzyme expression in tumor specimens and clinicopathological features was investigated. The results revealed that PON2 was significantly upregulated in BCC compared with controls, as well as in infiltrative lesions with respect to nodular lesions. Enzyme overexpression was also found in melanomas compared with nevi. In addition, a significant positive correlation was found between intratumor enzyme levels and important prognostic parameters such as Breslow thickness, Clark level, regression, mitosis, lymph node metastasis, pT, and pathological stage [44].

Subsequent immunohistochemical determinations of PON2 protein levels were performed in squamous cell carcinoma (SCC), the second most frequent NMSC, and surrounding healthy tissue, as well as in actinic keratosis (AK), the most common SCC precursor lesion. The results showed a significant increase in PON2 expression in SCC compared to control samples. Further comparison in terms of protein level was carried out in AK and tumors, distinguishing between less aggressive SCC (trunk and extremities) and more aggressive SCC (head and neck). As expected, precancerous lesions displayed lower PON2 immunoexpression with respect to SCC specimens, but interestingly, tumors in the head and neck region, associated with a less favorable prognosis, showed elevated enzyme levels compared with SCCs of the trunk and extremities [45].

In order to investigate the functions of the enzyme in melanoma, which accounts for less than 5% of all skin neoplasms but is responsible for most cutaneous malignancy-related deaths, shRNA-mediated gene silencing of PON2 was achieved in the A375 melanoma cell line and the effects on cell viability, proliferation, chemosensitivity, and ROS production were evaluated. The reported data indicated that enzyme knockdown led to significantly decreased cell proliferation and viability as well as enhanced sensitivity of A375 cells to CDDP. Moreover, under chemotherapeutic treatment, PON2 downregulation was associated with increased intracellular ROS formation [46].

### 2.3. PON2 and Gastrointestinal Cancers

Data obtained from TCGA, further confirmed by real-time PCR and immunohistochemistry, allowed the identification of enzyme upregulation in a large number of tumors obtained from patients with gastric cancer (GC) compared with normal tissue samples. Markedly high PON2 expression was mainly detected in tumors in the diffuse, infiltrating, and advanced clinical stages, as well as in patients with lymph node or distant metastasis, associated with remarkably short overall survival. In order to determine the enzyme’s biological role in GC cells, PON2 silencing was performed in the MKN45 and SGC-7901 GC cell lines, and the effects on tumor cell phenotype were investigated. The reported data clearly indicate that PON2 downregulation significantly inhibited cell viability, migration, and invasive capacity [47].

In a study by Nagarajan et al., the authors reported significant PON2 upregulation in association with PDAC, indicating the outstanding contribution of the enzyme to tumor development and spread. The data obtained from analyses of tumor cell lines led to the identification of the molecular mechanisms by which PDAC cells are positively affected by PON2 overexpression, thus promoting tumor progression. In the AsPC-1 PDAC cell line, PON2 transcription was found to be negatively regulated by tumor suppressor p53, and the constitutional lack of functional p53 exhibited by PDAC is mainly responsible for the elevated levels of enzyme detected in this neoplasm. PON2 was found to interact with GLUT1 glucose transporter, thus facilitating monosaccharide translocation into PDAC cells. This condition greatly increases the efficiency of both glucose uptake and metabolism. PON2-mediated metabolic reprogramming allows PDAC cells to optimally satisfy the demand for energy that is required for rapid and efficient cell proliferation. In addition, PON2 is able to inhibit anoikis, an apoptotic cell death program promoted by the activation of AMPK, which in turn is induced upon starvation. All of this evidence clearly demonstrates the enzyme’s ability to confer an adaptive advantage on PDAC cells, as well as how different aspects of cancer cell aggressiveness are impacted by PON2 [38,48].

### 2.4. PON2 and Genitourinary Cancers

The mRNA and protein levels of PON2 were found to be significantly increased in tumors compared with normal tissue samples obtained from bladder cancer (BC) patients. The analysis of enzyme expression was also extended to exfoliated urinary cells from subjects with BC and healthy controls, as PON2 mRNA levels are significantly inversely related to the tumor stage. In addition, the induction of PON2 overexpression in the T24 BC cell line led to markedly enhanced cell proliferation and resistance to oxidative stress, as demonstrated by the ROS production observed upon treatment with tert-butyl-hydroperoxide, a pro-oxidant [49]. In order to explore the enzyme’s role in malignant bladder cell transformation and BC progression, PON2 silencing and upregulation were induced in the T24 BC cell line and the impact on different aspects related to aggressive cellular phenotype was investigated. The experimental activity associated with the modulation of PON2 expression clearly demonstrated the enzyme’s capacity to positively affect both cell viability and migration. Further analyses were focused on evaluating the contribution of PON2 to sensitizing BC cells to the effect induced by chemotherapeutic treatment in terms of proliferative capacity, ROS production, and activation of caspase-3 and -8, which execute apoptosis. The results indicated that under treatment with CDDP and gemcitabine, T24 cells with downregulated PON2 exhibited significantly decreased cell viability, together with enhanced ROS-related oxidative stress and caspase activation, and these effects were totally reversed when enzyme overexpression was induced [50].

A study by Bacchetti et al. investigated the effect induced by the extract of flower buds of *C. spinosa* subsp. *rupestris* in terms of cell proliferation, intracellular ROS production, and induction of PON2 expression in T24 cells and the human immortalized urothelial UROtsa cell line, used as control. The data obtained demonstrated that flower bud extract, which had a high content of polyphenols and glucosinolates, exerted a greater antiproliferative effect, leading to increased ROS release and PON2 overexpression in T24 compared to UROtsa cells [51].

The cytotoxic effect of gossypol (GP), a phytochemical compound, and zoledronic acid (ZA), a nitrogen-containing bisphosphonate, was evaluated in the human androgen receptor-negative and drug-resistant DU-145 prostate cancer cell line, an experimental cellular model for studying the behavior of aggressive metastatic human prostate carcinoma. Interestingly, the results indicated that both GP and ZA had significant antiproliferative capacity when sequentially administered, used alone or in combination, in this case exhibiting a synergistic effect. In addition, drug treatment also led to increased cellular apoptosis in terms of DNA fragmentation and caspase-3/-7 enzyme activity, and inhibited expression of anti-apoptotic proteins, including PON2 [52].

Among gynecologic cancers, PON2 mRNA expression was found to be upregulated in a larger number of tumor tissues compared to their normal counterparts obtained from women with ovarian cancer (OC) [32]. Subsequent immunohistochemical analyses were carried out to investigate the protein level expression in OC tissue specimens and normal tissue samples. The reported data showed that the PON2 enzyme was significantly overexpressed in stage I and II tumors compared with matched normal-looking tissues, whereas no difference in protein expression was detected in stage III and IV lesions. Further analyses, focused on determining the mechanism of action of PON2 in OC cells, were carried out using an in vivo mouse model. The mouse ID8 OC cell line, which overexpresses PON2, and control cells were subcutaneously injected into the flanks of C57BL/6J nude mice, and tumor formation was monitored. The molecular mechanisms promoting PON2-mediated OC cell proliferation upon injection were also investigated. The results showed that enzyme upregulation significantly inhibited in vivo tumor formation. This effect was induced by PON2’s ability to decrease OC cell proliferation by inhibiting insulin-like growth factor 1 (IGF-1), suggesting that it potentially has a tumor-suppressing role [53].

### 2.5. PON2 and Leukemia

As reported in previous studies, PON2 upregulation was found in cohorts of pediatric ALL patients with a poor prognosis and in patients with CML resistant to targeted imatinib-based therapy [33,34,35]. In particular, microarray analysis demonstrated that PON2 was identified among the overexpressed genes in pediatric ALL patients experiencing adverse outcomes [34].

In vitro investigations showed that the enzyme is overexpressed in the Lama84 and KCL22 CML cell lines resistant to imatinib compared with sensitive counterparts and that PON2 upregulation is mediated by Lef-1 through activation of the Wnt/GSK3β/β-catenin pathway [41]. In research focused on clarifying the potential contribution of PON2 to imatinib resistance in CML patients, data reported in a study by Witte et al. demonstrated that enzyme upregulation in the Bcr-Abl-positive K562 CML cell line protected the cells from injury and death induced by apoptosis associated with imatinib, acting as a Bcr-Abl tyrosine-kinase inhibitor. Conversely, PON2 silencing was able to reverse these effects, increasing cell sensitivity to imatinib by enhancing apoptosis [32].

Further studies on ALL revealed that PON2 mRNA levels were significantly higher in B-ALL compared with normal pre-B cells, with elevated enzyme expression being a potential predictor of unfavorable clinical outcomes in both pediatric and adult B-ALL patients. In particular, increased overall survival and relapse-free survival were significantly associated with low PON2 expression. Therefore, it was speculated that the enzyme has a role in normal B cell development and during the leukemogenesis process. PON2 deletion in two murine B-ALL cell lines markedly compromised their cell proliferation and colony formation ability, and reduced leukemia initiation and development upon transplantation in mice in vivo. Similarly, CRISPR-Cas9-mediated enzyme knockout in human B-ALL cells significantly decreased proliferation and survival. By contrast, reduced PON2 expression in mice did not result in any relevant phenotypic changes/differences between pro-B, early pre-B, immature, and mature B cells, confirming that even if the enzyme is crucial for leukemogenesis in B-ALL, it does not affect normal B cell development. PON2 promotes leukemogenesis in murine and human B-ALL cells by facilitating glucose uptake through interaction with the GLUT1 transporter, and by increasing ATP formation. In particular, the function of increasing glucose transport in cells is based on PON2’s capacity to block the interaction between GLUT1 and its inhibitor, represented by the integral membrane protein stomatin (STOM) [54].

Glucocorticoids, including dexamethasone (Dex), are used during B-ALL chemotherapy regimens due to their ability to inhibit the glucose transport system, even if resistance phenomena are often acquired. Given PON2’s role as a metabolic gatekeeper related to glucose transport, the association between enzyme expression and the efficacy of Dex treatment was explored. The reported data demonstrated that PON2 deficiency sensitizes mouse and human B-ALL cells to the effects of Dex [54]. A further study revealed that PON2 levels were significantly higher in peripheral blood lymphocytes obtained from ALL patients compared to controls, and in Dex-resistant compared to Dex-sensitive B-ALL patients. Related in vitro investigations demonstrated that the enzyme was able to inhibit apoptosis. Moreover, PON2 silencing in Dex-resistant B-ALL cells led to reduced tumor growth following xenograft implantation in immunodeficient mice [55].

### 2.6. PON2 and Other Cancers

PON2 protein expression was found to be increased in glioblastoma multiforme (GBM) compared to normal brain tissue. Treatment with valproic acid (VPA) induced cell cycle arrest, increased ROS production, and led to decreased PON2 levels in human U87, GBM8401, and DBTRG-05MG GBM cell lines. VPA treatment exerted a negative stimulation effect on enzyme expression at the transcriptional level, mediated by the interaction between VPA and a response element located on the PON2 promoter. Interestingly, the induction of enzyme overexpression reversed the above effects, leading to decreased intracellular ROS [56].

In vitro analysis of thyroid cancer cell lines revealed that LINC00488 long non-coding RNA (lncRNA) was markedly overexpressed, promoting several aspects of tumor cell phenotype, such as proliferation, migration, invasion, and resistance to apoptosis. These effects were mediated by the capacity of lncRNA to interact with and bind to miR-376a-3p, inducing its downregulation. Interestingly, as PON2 is a miR-376a-3p target gene, LINC00488 is responsible for enhanced expression of the enzyme detected in thyroid cancer [57].

Based on previously reported evidence demonstrating PON2 upregulation in lung cancer [58], further studies were conducted to investigate lung cancer tumorigenesis, both in vitro and in vivo. Induced enzyme knockdown and knockout significantly impaired cell proliferation and promoted cell cycle arrest in murine Lewis lung carcinoma (LCC) cell line and human A549 and NCI-HI1299 lung adenocarcinoma cells. Subsequent investigation of the impact of PON2 on cellular bioenergetics and metabolism revealed that enzyme loss markedly reduced glycolysis, tricarboxylic acid cycle activity, and de novo pyrimidine nucleotide biosynthesis [39]. As previously reported, also in lung cancer, PON2 overexpression was found to counteract programmed cell death by balancing ROS production. In A549 cells, enzyme knockdown allowed significant ROS release, leading to the induction of proapoptotic CHOP expression via the JNK signaling pathway [32].

PON2 immunohistochemical expression was also evaluated in several molecular subtypes of breast cancer: luminal A, luminal B, luminal B HER2+, HER2+, and triple-negative breast cancer (TNBC). Enzyme levels were significantly increased in infiltrating neoplasms compared to surrounding healthy tissue related to the luminal A, HER2+, and TNBC subtypes. Since TNBC is highly aggressive, has limited therapeutic options, and is chemoresistant, further investigation was focused on exploring PON2’s involvement in the molecular mechanisms underlying cell proliferation and sensitivity to drug treatment affecting this tumor form. The data obtained demonstrated that enzyme silencing was associated with reduced cell proliferation and an increased cytotoxic effect induced by chemotherapy compounds in MDA-MB-231 TNBC cells, suggesting a promising role for PON2 as a molecular target for TNBC treatment [59].

## 3. Conclusions

This review, focused exclusively on PON2, provides a general, complete, updated overview of the bulk of studies published in the last two decades describing how enzyme involvement in molecular processes and cellular events promotes malignant transformation and features tumor cell aggressiveness.

**Figure 2:**
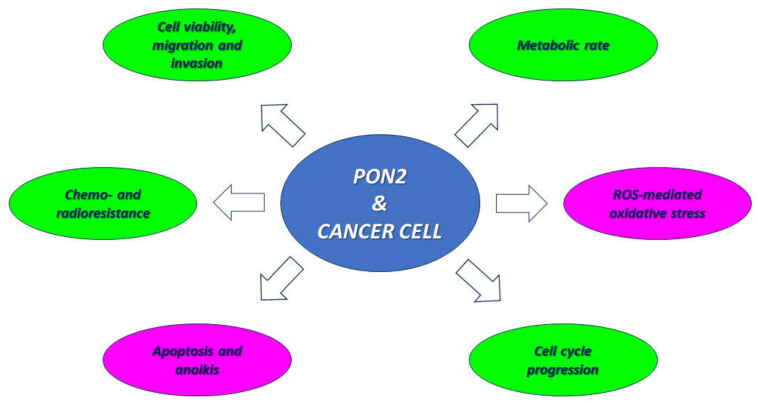
Role of PON2 in cancer cell phenotypes. Molecular, biochemical, and cellular events and phenotypic traits associated with tumor cell aggressiveness in which PON2 enzyme involvement was demonstrated. Green and pink ovals indicate enhanced and inhibited pathways following PON2 overexpression, respectively.

Consistent with the data reported in these studies [60,61,62], the PON2 protein seems to exert a protective effect on tumor cells against anti-neoplastic strategies, including radiotherapy, chemotherapy, and targeted drugs, partly by promoting cell proliferation and mainly by counteracting oxidative stress and apoptosis-induced cell death. In this regard, the enzyme overexpression observed in several kinds of solid and blood tumors confers an adaptive advantage on cancer cells. In addition to inhibiting apoptosis and promoting cell cycle progression, PON2 appears to have a strong ability to increase cellular metabolic activity by enhancing glycolysis as well as the tricarboxylic acid cycle and nucleotide biosynthesis (Figure 2).

Altogether, these data strongly suggest that the enzyme may be a candidate as an interesting molecular target in the development of effective strategies to manage/treat/cure cancers. In this regard, the availability of homogeneous purified recombinant protein in the future will allow us to determine its tridimensional structure and hopefully disclose crucial aspects related to its catalytic mechanism of action. Based on these findings, compounds that inhibit PON2 enzyme activity could be designed, synthesized, and assayed in order to explore their potential use as novel and promising anti-cancer therapeutic agents.
